# Autonomous Addition of Agents to an Existing Group Using Genetic Algorithm

**DOI:** 10.3390/s20236953

**Published:** 2020-12-05

**Authors:** Sabyasachi Mondal, Antonios Tsourdos

**Affiliations:** School of Aerospace, Transport and Manufacturing (SATM), Cranfield University, Cranfield MK430AL, UK; a.tsourdos@cranfield.ac.uk

**Keywords:** distributed control, optimal topology, consensus, two-dimensional genetic algorithm, autonomous mission, BVLOS

## Abstract

This paper presents an idea of how new agents can be added autonomously to a group of existing agents without changing the existing communication topology among them. Autonomous agent addition to existing Multi-Agent Systems (MASs) can give a strategic advantage during the execution of a critical beyond visual line-of-sight (BVLOS) mission. The addition of the agent essentially means that new connections with existing agents are established. It is obvious that the consensus control energy increases as the number of agent increases considering a specific consensus protocol. The objective of this work is to establish the new connections in a way such that the consensus energy increase due to the new agents is minimal. The updated topology, including new connections, must contain a spanning tree to maintain the stability of the MASs network. The updated optimal topology is obtained by solving minimum additional consensus control energy using the Two-Dimensional Genetic Algorithm. The results obtained are convincing.

## 1. Introduction

In recent times, drones have been found to be useful for many applications such as in agriculture, delivery services, transportation, and search and rescue. Many complex operations involved in these applications must be executed by Multi-Agent Systems (MASs) in which the agents perform tasks cooperatively. Irrespective of the application, the agents must achieve a consensus to continue operations cooperatively. A considerable amount of work on the consensus problem has been reported in the literature. Other examples of collective behaviour of cooperative platforms are formation control [1,2], Synchronization [3,4], and consensus tracking [5].

In the majority of applications, drones are usually operated by remote pilots. Therefore, the range of operation is limited to visual line-of-sight (VLOS). However, the demand for beyond VLOS (BVLOS) operation is increasing rapidly. The implementation of autonomy is important to reduce the dependency on the remote pilot and to achieve BVLOS operation. Therefore, autonomous operation of drones is tan important area od research. It can be noted that autonomous operation of MASs depends on consensus building and information sharing among the agents, which msut be appropriately connected (i.e., have a well defined communication topology). The consensus is achieved by designing various consensus protocols. Generally, these protocols are designed considering the communication topology among agents, agent dynamics, information on the states of neighbours, and communication-related issues such as switching topology, communication delay, and noise. In many research works, the consensus protocols have been designed considering these factors. A few of them are mentioned here. In [6], the distributed consensus tracking problem was addressed for multi-agent systems with Lipschitz-type node dynamics. The consensus tracking problem has been solved with changing the topology among followers. [5] describes the consensus tracking problem where the agents track a time-varying reference state. The agents have second-order nonlinear dynamics. The communication topology is considered to change with time. [7] addressed the consensus problem for heterogeneous multi-agent systems with first- and second-order dynamics. The agents are subjected to probabilistic link failure. The random nature of these failures is represented using a Bernoulli probability sequence. [8] considers directed and switching communication topology for solving the leaderless consensus problem of multi-agent systems with Lipschitz nonlinear dynamics. In [9], the average consensus problem in distributed multi-agent systems was discussed. The authors considered undirected and connected graphs to represent the communication topology among the agents. They also considered time delays in the communication channel. The work in [10] focused on asynchronous consensus of multiple agents with double-integrator dynamics. It assumed arbitrary sampling intervals and communication delays. In [11], the consensus problem of agents with single integrator dynamics was considered. However, the communication topology was assumed to be fixed. The work in [12] described a mean square consensus protocol for multi-agent systems with linear dynamics. The communication topologies among the agents were fixed, and channel noise was present. [13] describes the consensus of multi-agent systems with communication noise considering linear dynamics. A consensus algorithm for multi-agent communication with noise can be found in [14]. Ref. [15] also reports consensus with communication noise in multi-agent systems (MASs) with stochastic dynamics.

**Figure 1:**
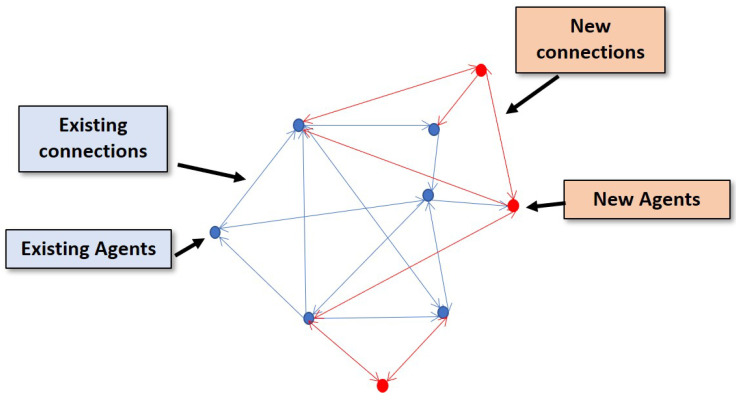
Pictorial representation of the problem. The new agents need to be added by establishing new connections with existing agents while maintaining the current topology.

In a real-world scenario, the objectives of an ongoing mission change and more agents need to be involved along with the existing group. To make these additional agents part of the existing network, new connections must be established with them. Moreover, for a BVLOS mission, the connections must be established autonomously without the involvement of a remote pilot. Therefore, the autonomous establishment of new connections between new and existing agents or autonomous agent addition is crucial in the context of BVLOS operations. In this paper, the authors present an approach for the autonomous addition of new agents to an existing MASs in an optimal manner, which is extremely important to design an energy-efficient autonomous UAV platform for BVLOS operations (Figure 1).

One of the important advantages of this approach is that the agent addition will not affect the existing topology among the agents, i.e., no need to reconfigure or modify the existing topology. Reconfiguration of the existing topology requires the cancellation of the existing one and new connections are established among all the agents (including new agents) according to a new topology. This is impossible because cancelling and establishing new connections in the middle of a mission may lead to instability among the agents and loss of agent information. The optimal manner means the addition of agents would be in such a way that the consensus of all the agents (including the new agents) can be achieved by spending the minimum additional cost, i.e., control energy. It has been mentioned earlier that the consensus of the agents depends on the communication topology. This is due to the fact that the consensus protocol is designed considering the communication topology, i.e., the consensus control expression (for example, Equation (11)) contains the communication status among the agents (described by the matrix A). It is important to note that the performance of these consensus protocols is measured by consensus control energy, which is the amount of control energy (a quadratic function of the consensus protocol) that the agents should spend to achieve the consensus). The addition of the agents essentially means that new connections with existing agents are established. It is obvious that the consensus control energy increases as the number of agent increases, considering a specific consensus protocol. The objective of this work is to establish the new connections in such a way that the increase n the consensus energy due to the new agents is minimal. In other words, the new connections will be established in a manner such that the consensus control energy of all the agents (including new agents) increases as little as possible compared to the cost required for the consensus of existing agents.

The contribution in this work is summarized as follows.

- Autonomous agent addition to an existing group of agents is a new idea and has not been reported in the literature (to the best of authors’ knowledge). It is an important operation when an ongoing mission demands more agents. The idea helps to build a distributed architecture which is appropriate for BVLOS operations. The agents can operate independently if more agents are required to join the group. There is no need to depend on remote pilots.
- The existing communication topology is not modified. This is important because the existing agents remain connected during the flight. Therefore, network stability is assured. The minimum additional consensus control is required. This is necessary to ensure a minimum number of new connections should be established. In addition, the MASs can operate for a longer duration, which is beneficial for a mission.
- A bio-inspired optimization technique is used to solve the problem which is new in this context. A new crossover and mutation variety is proposed.

The remainder of the paper is organized as follows. The problem description is given in Section 2. In Section 3, preliminaries are presented. The solution method is discussed in Section 4. The results are shown in Section 5. Finally, the conclusion is given in Section 6.

## 2. Problem Description

**Figure 2:**
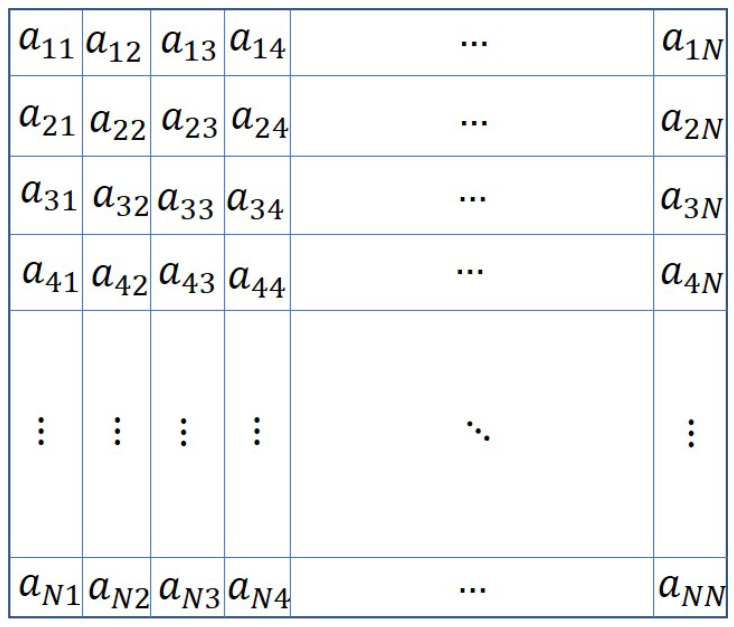
Adjacency matrix *A* defining the existing topology.

The problem can be explained with the help of an example. Let us consider a group of *N* agents assigned to a mission. They are connected by a communication topology which is shown by an adjacency matrix $A=[a_{ij}];\;i,j=1,2,\ldots ,N$ is shown in Figure 2. The elements $a_{ij}=0$ or 1,∀i≠j and $a_{ij}=0,\forall i=j$.

Let us consider a situation where the mission demands more agents and a group of *n* new agents (not connected to each other) are required to add to the existing agents. The agents can be added by establishing connections among the existing and new agents, i.e., reconfiguring or modifying the existing communication topology. There are two possible ways to reconfigure the existing topology to accommodate the new agents. The first one is to configure a fresh topology among all the agents. The second one is to connect the new agents to existing agents arbitrarily. In the first way, the existing topology is cancelled, and new connections are established among all the agents (including new agents) according to a new topology. This is not possible because cancelling and establishing new connections may lead to instability of the existing graph topology. This may also cause the loss of agent information.

**Figure 3:**
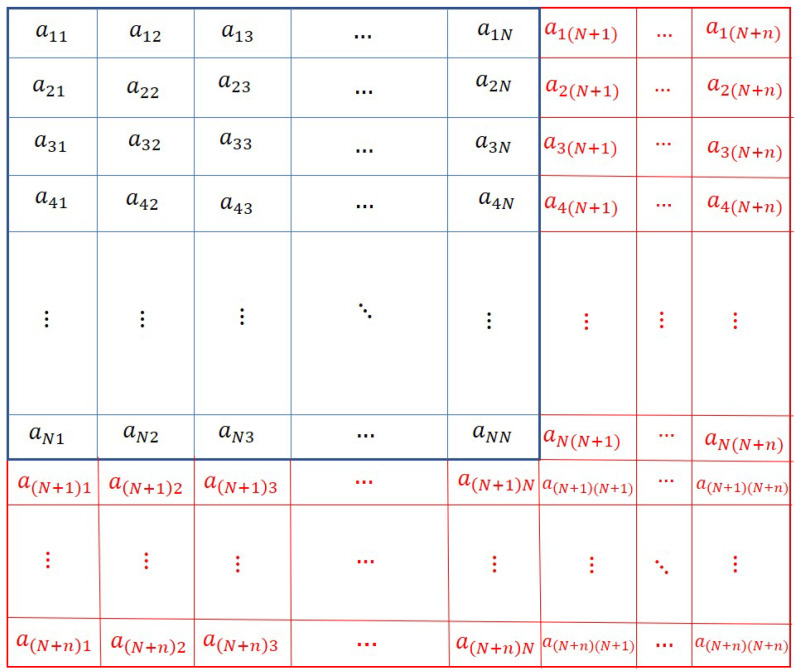
Modified Adjacency matrix $A_{mod}$ defining the modified topology which includes new agents.

In the second way, new connections are established among the new and existing agents. The pictorial representation is given in Figure 3.

Comparing Figure 2 with Figure 3, it can be observed that the new connections among the new and the existing agents are denoted by the values of the elements $a_{ij},\;\left(i=N+1,\ldots ,N+n;\;j=1,2,\ldots ,N+n\right)$ and (i=1,…,N;j=N+1,…,N+n). These elements are shown in red in Figure 3. The binary values of these elements (except the diagonal elements) are appended to the existing adjacency matrix *A* to obtain the modified adjacency matrix $A_{mod}$. However, the assignment of values of the elements (in red colour) corresponding to new agents, cannot be done randomly. The elements should be assigned such that the resulting topology contains a spanning tree, and the consensus can be achieved. Moreover, the resulting topology may not be optimal. The optimal topology is the topology in which the agents if connected, require minimum control energy to achieve the consensus.

**Figure 4:**
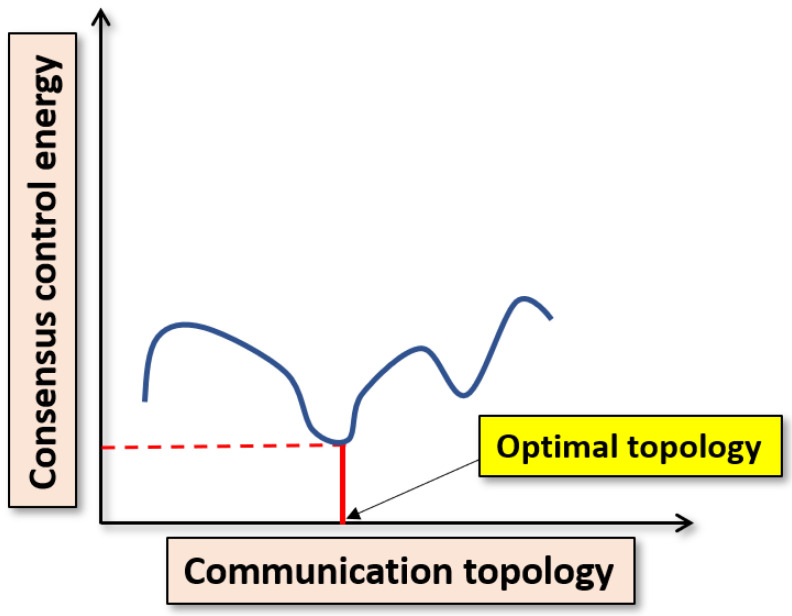
Problem description: Control energy varies with communication topology. (using any consensus protocol).

In the context of control energy about the consensus of MASs, it is important to note that the same consensus protocol can produce different control energies for the agents if the topology varies. Therefore, the control energy can be minimized with respect to the topologies. The topology corresponding to the minimum energy is addressed as the optimal topology (see Figure 4 for details). A similar problem has been addressed in [16,17,18]. In these papers, the Linear Quadratic Regulator (LQR) was used to obtain a complete graph for the homogeneous systems. A similar problem is presented in [19], where heterogeneous agents are considered. The optimal topology obtained is a star graph which shows the direct links between the leader and the followers. In [20], the authors presented the optimal topology problem and solved it using a Two-Dimensional Genetic Algorithm (2D-GA), which relaxes the requirement of direct connection among the leader and the followers.

It is important to note that an increase in the number of agents in a MASs will result in an increase in the consensus control energy. In this problem, let us consider the consensus control of ith agent is $u_{i}$, consensus energy of the existing *M* agents is $J=\sum_{i=1}^{M}u_{i}^{T}u_{i}$, and the required unknown consensus control energy for the modified MASs (including the new agents) be $J_{mod}$.

**Figure 5:**
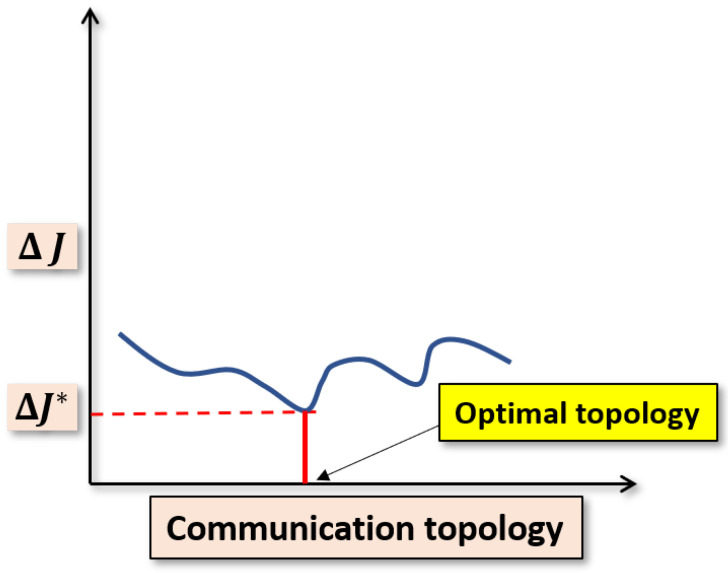
Problem description: Additional consensus control energy ΔJ varies with communication topology.

It is obvious that the consensus control energy of the agents will increase with the increase in the number of agents, i.e., $J_{mod}>J$. The objective of this paper is to minimize the difference $\Delta J=J_{mod}-J$ (additional consensus control energy). Since *J* is constant, minimization of ΔJ is equivalent to minimizing $J_{mod}$ to find the minimum $J_{mod}$, i.e., $J_{mod}^{*}$ such that the difference ΔJ becomes minimal, i.e., $\Delta J^{*}=J_{mod}^{*}-J$. The problem description is given in Figure 5.

## 3. Preliminaries

The preliminaries required for the research work are presented in the following section.

### 3.1. Consensus of Agents

The consensus of MASs on a communication network is discussed in this section. The definition of the consensus is given as follows. efinition

**Definition** **1.** *Let us consider a MASs with N agents, where $X_{i},\;\left(i=1,2,3,\ldots ,N\right)$ denotes the states of the ith agent. The MASs will achieve the consensus if $\|X_{i}-X_{j}\|\to 0,\forall i\neq j$ as t→+∞.*

The primary goal of designing a consensus protocol is to minimize the error in similar states of each individual agent with its neighbour by exchanging information among them through the communication network, which is designed and explained by graph theory.

### 3.2. Graph Theory

The communication among the agents is designed using graph theory. The networked MASs is represented by a weighted directed graph written by G={V,E}. The vertices V={1,2,…,N} of the graph denotes the agents and the set of edges is denoted by E⊆V×V represents the communication among the agents. $e_{ij}$ denotes the information flow along the edge from *j* to *i*. The neighbour of agent *i* is denoted by $N_{i}=\left\{j\in V:\left(i,j\right)\in E\right\}$. The Adjacency matrix $A=\left[a_{ij}\right]\in {ℜ}^{N\times N}$ denotes the connectivity among the nodes or agents. $a_{ij}$ denotes the elements of *A*. There is no self loop in the graph. This fact is expressed by selecting the diagonal elements of the adjacency matrix *A* as zero, i.e., i∈V, $a_{ii}=0$. The off-diagonal elements, i.e., ∀$i\neq j,e_{ij}\in E$, $a_{ij}\in {ℜ}^{+}$ represent the weight associated to edge $e_{ij}$, while $a_{ij}=0$ otherwise. The degree matrix is denoted by $D\in {ℜ}^{N\times N}=diag\left\{d_{1}\;d_{2}\;\ldots d_{N}\right\}$, where $d_{i}=\sum_{j\in N_{i}}a_{ij}$. The Laplacian matrix is written as L=D−A. All the matrices describe the connections and properties of the Consensus among MASs.

### 3.3. Distributed Nonlinear Dynamic Inversion (DNDI) Controller for Consensus of MASs

There exist consensus protocols for nonlinear systems, as discussed earlier. However, in this paper, a new consensus protocol is used, which is designed using Nonlinear Dynamics Inversion (NDI). The derivation of NDI-based control for MASs, named as DNDI, is presented in this section. In case of consensus of MASs, the reference signal or desired output of each agent is the outputs of its neighbours. The dynamics of the ith agent is given by

(1) $$\begin{array}{ccc} {\dot{X}}_{i} & = & f\left(X_{i}\right)+g\left(X_{i}\right)U_{i} \end{array}$$

(2) $$\begin{array}{ccc} Y_{i} & = & h(X_{i}) \end{array}$$

where $X_{i}\in {ℜ}^{m}$ is state and $Y_{i}\in {ℜ}^{n}$ is output. The mathematical derivation of DNDI is shown below.

First, the error associated with the scalar output is obtained. The error in output of ith agent can be written as

(3) $$e_{i}=\sum_{j\in N_{i}}a_{ij}\left(Y_{i}-Y_{j}\right)$$

where *j* denotes the jth agent of ith agent’s neighbourhood $N_{i}$. The Equation (3) can be simplified as follows

(4) $$e_{i}=d_{i}Y_{i}-a_{i}Y$$

where

$$d_{i}=\sum_{j}a_{ij}\in ℜ,\;a_{i}=\left[a_{i1}\;a_{i2}\ldots \;a_{iN}\right]\in {ℜ}^{N}$$

and

$$Y=\left[\begin{array}{c} Y_{1}\\ Y_{2}\\ \vdots \\ Y_{N} \end{array}\right]\in {ℜ}^{N}$$

The error in Equation (4) is written for vector output of ith agent i.e., $Y_{i}\in {ℜ}^{n};n>1$ as

(5) $$e_{i}=\bar{d}Y_{i}-\bar{a}Y$$

where $\bar{d}=\left(d_{i}⊗I_{n}\right)\in {ℜ}^{n\times n}$, $\bar{a}=\left(a_{i}⊗I_{n}\right)\in {ℜ}^{n\times nN}$, and $Y\in {ℜ}^{nN}$. $I_{n}$ is n×n identity matrix. ‘⊗’ denotes the kroneker product. The kroneker product of $A=[a_{ij}]$ and *B* is given by

$$A⊗B=[a_{ij}B]\;\forall i,j$$

We define a Lyapunov function $V_{i}$ as follows

(6) $$V_{i}=\frac{1}{2}e_{i}^{T}e_{i}$$

Differentiating Equation (6) yields

(7) $${\dot{V}}_{i}=e_{i}^{T}{\dot{e}}_{i}$$

According to the Lyapunov stability theory, let the time derivative of the lyapunov function be

(8) $${\dot{V}}_{i}=-e_{i}^{T}Ke_{i}$$

where $K\in {ℜ}^{n\times n}$ is a positive definite diagonal matrix. The expression of ${\dot{V}}_{i}$ in Equations (7) and (8) are equated to obtain

(9) $$e_{i}^{T}{\dot{e}}_{i}=-e_{i}^{T}Ke_{i}$$

Equation (9) is simplified as follows

(10) $${\dot{e}}_{i}+Ke_{i}=0$$

Differentiation of Equation (5) yields

(11) $$\begin{array}{ccc} {\dot{e}}_{i} & = & \bar{d}{\dot{Y}}_{i}-\bar{a}\dot{Y}\\ & = & \bar{d}\left[f\left(X_{i}\right)+g\left(X_{i}\right)U_{i}\right]-\bar{a}\dot{Y} \end{array}$$

Substitution of the expressions for $e_{i}$ and ${\dot{e}}_{i}$ in Equation (10) gives

(12) $$\bar{d}\left[f\left(X_{i}\right)+g\left(X_{i}\right)U_{i}\right]-\bar{a}\dot{Y}+K\left(\bar{d}Y_{i}-\bar{a}Y\right)=0$$

The expression of control $U_{i}$ for ith agent is obtained by simplifying Equation (12) as follows

(13) $$U_{i}={\left(g\left(X_{i}\right)\right)}^{-1}\left[-f\left(X_{i}\right)+{\bar{d}}^{-1}\left(\bar{a}\dot{Y}-K\left(\bar{d}Y_{i}-\bar{a}Y\right)\right)\right]$$

The consensus control energy is given by $J=\sum_{i=1}^{N}u_{i}^{T}u_{i}$.

It is clear that the control expressions of conventional NDI are different from what is obtained for the consensus of MASs. This control expression in Equation (13) was used to generate the results in this paper.

## 4. Solution Method: Two-Dimensional Genetic Algorithm (2D-GA)

A genetic algorithm (GA) is a class of bio-inspired algorithms that can produce the optimal or near-optimal solution of complex optimization problems in a reasonable time. It was first proposed by Holland [21], and inspired by Darwin’s principle of survival of the fittest. The possible solution of an optimization problem is encoded in a chromosome which consists of an array of bits called genes. The individual chromosome is evaluated by a fitness function. A genetic population consists of a finite number of chromosomes. The chromosomes of the new population are generated by the application of genetic operations such as crossover, mutation, and reproduction on the present population. The new population optimizes the fitness function and thus provides an improved solution. The solution thus approaches the optimal solution over several generations. There exist many applications of GA viz. optimization [22,23], machine learning [24], neural networks [25], fuzzy logic controllers [26], identification [27], fault diagnosis [28], path planning [29], consensus [30], and financial market [31].

The optimization problem mentioned in the previous section cannot be solved by convensional GA. The solution to this problem can be obtained using a two-dimensional genetic algorithm. The chromosome of 2D-GA is a matrix which is appropriate to represent an adjacency matrix [20]. Therefore, in this paper, the adjacency matrix is considered as a chromosome. Depending on the application, the two-dimensional chromosome represents a different solution. For example, the time table or schedule is considered as a 2D chromosome in a flight scheduling problem in [32]. Also, it is used in the packing problem [33], which aims to obtain a high packing density. The two-dimensional chromosome representation is discussed in the following section.

### 4.1. Two-Dimensional Chromosome Representation

The chromosome for this problem is the modified adjacency matrices $A_{mod}$, which is discussed in Section 3. In [20], the authors considered the adjacency matrix as a 2D chromosome. An example of such a chromosome is given in Figure 1. As explained in Section 3, the agent addition problem requires the adjacency matrix to be modified to represent the new connections among the new and existing agents. The modified adjacency matrix is shown in Figure 2. The modified adjacency matrix preserves the properties of the adjacency matrix and serves as guess solution or chromosomes for 2D-GA. The population generation is shown in the following section.

### 4.2. Population Generation

It is clear that the chromosomes are square as the adjacency matrix is square. Let us consider, there are *N* agents in the existing MASs, and *n* new agents need to be added. The adjacency matrix or existing communication topology is denoted by $A\in {ℜ}^{N\times N}$.

**Figure 6:**
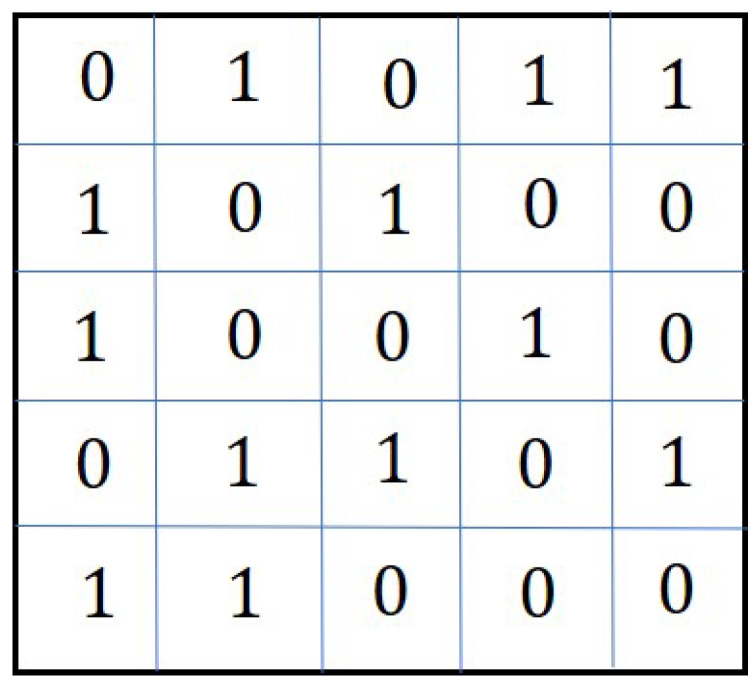
Adjacency matrix showing the existing topology.

These new connections among existing and new agents are appended to the existing topology *A* to obtain the modified adjacency matrix or modified 2D chromosome $A_{mod}$. The kth chromosome $A_{mod_{k}},\;k=1,2,\ldots ,N_{p}$ can be generated using Algorithm 1, where $N_{p}$ denotes the population size. The algorithm can be explained with the help of an example. Let us consider, N=5 and n=3 and the existing topology as shown in Figure 6.

**Figure 7:**
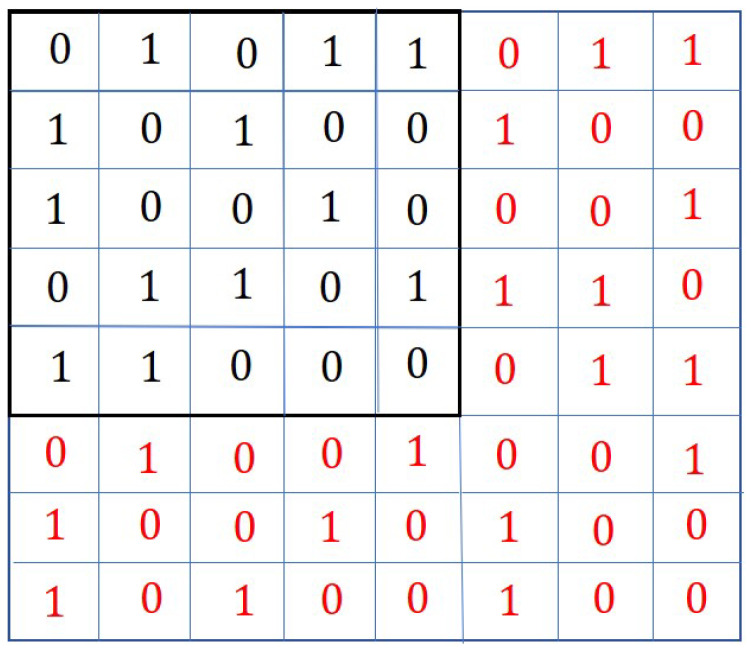
Adjacency matrix showing the existing topology.

The algorithm produces the population Pop whose kth chromosome $A_{mod_{k}}$ is shown in Figure 7.

The 6–8th rows and 1–8th columns are filled up in a random manner depending on the random value of the variable *x*. Similarly, the 1–5th rows and 6–8th columns are filled up. The new diagonal elements are zero. The population thus generated is subjected to crossover and mutation operations which are described in the following sections.
**Algorithm 1** Initial Population Generation.**for**k=1 to $N_{p}$ **do**  **for** i=N+1 to N+n **do**      **for** j=1 to N+n **do**      x← random number x∈(0,1)      **if** x>0.5 **then**
          A(i,j)←1
      **else**
          A(i,j)←0
      **end if**
      **if** i=j **then**
          A(i,j)←0
      **end if**
      **end for**
  **end for**
  **for** i=1 to *N* **do**      **for** j=N+1 to N+n **do**      x← random number x∈(0,1)      **if** x>0.5 **then**
          A(i,j)←1
      **else**
          A(i,j)←0
      **end if**
      **end for**
  **end for**
  $A_{mod_{k}}\leftarrow A$
  $Pop\left(:,:,k\right)\leftarrow A_{mod_{k}}$
**end for**

### 4.3. Crossover

There are a few Crossover methods that exist in the literature. Some of these methods are Multipoint Crossover [34], Uniform Crossover [35], One-Point Crossover [36], and Substring Crossover [36]. More crossover methods can be found in [37]. The crossover method mentioned in [32] is adopted in this work. These methods are presented in algorithmic form. The crossover algorithm is modified (Algorithm 2) to apply it for the modified chromosome (including new agents), as shown in Figure 3.
**Algorithm 2**Crossover().Generate random integer $r_{1}\in \left(N,N+n\right)$Generate random integer $r_{2}\in \left(1,N+n\right)$$Block1_{P1}$$\leftarrow Parent1(r_{1},r_{2}:N+n)$$Block2_{P1}$$\leftarrow Parent1(r_{1}+1:N+n,1:N+n)$$Block1_{P2}$$\leftarrow Parent2(r_{1},r_{2}:N+n)$$Block2_{P2}$$\leftarrow Parent2(r_{1}+1:N+n,1:N+n)$$Block1_{P1}⇌Block1_{P2}$ and $Block2_{P1}⇌Block2_{P2}$

**Figure 8:**
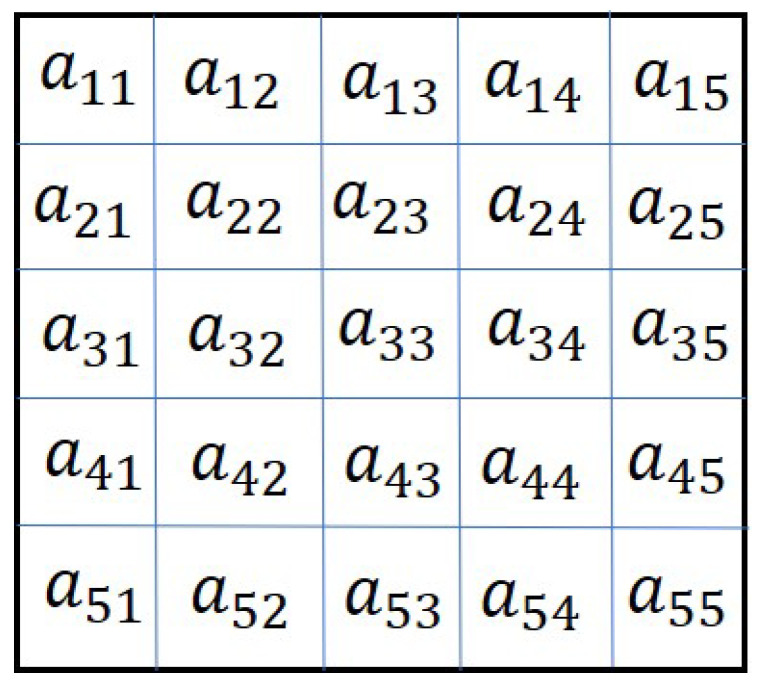
Adjacency matrix showing the existing topology.

The algorithm can be explained using an example. Let us consider N=5 and n=3. A general example of existing topology $A=[a_{ij}],i,j=1,2,\ldots ,5$ is shown in Figure 8.

**Figure 9:**
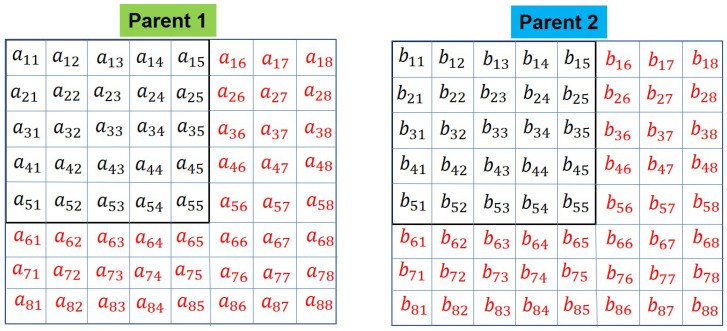
Adjacency matrix showing the modified topology.

The modified topology or 2D parent chromosomes are denoted by ‘Parent 1’, and ‘Parent 2’. They are shown in Figure 9.

**Figure 10:**
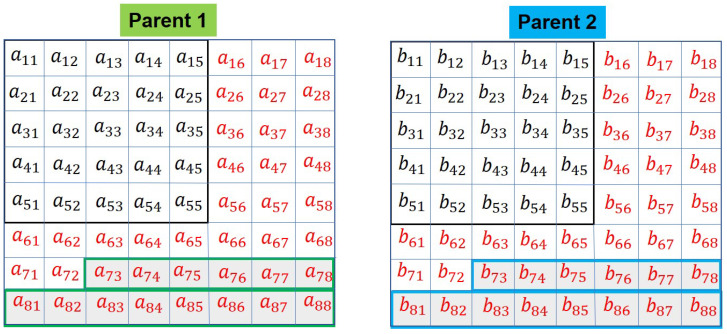
Parents: horizontal crossover, selected genes are shown in green and blue border.

According to the Algorithm 2, the random integers $r_{1}$ and $r_{2}$ are obtained as $r_{1}=7,r_{2}=3$. The selected elements around the crossover point are shown in the shaded area with a green outline in Parent 1 and blue outline in Parent 2. They are shown in Figure 10.

**Figure 11:**
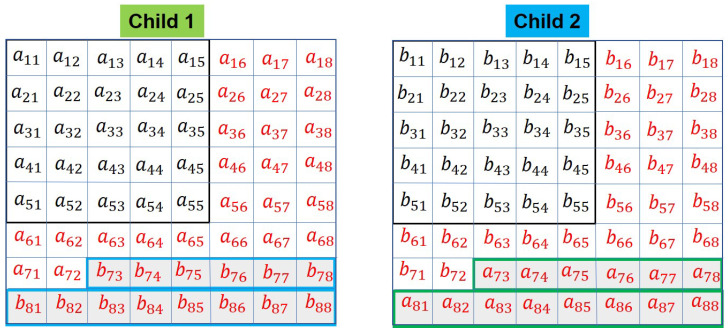
Children: horizontal crossover, selected genes are exchanged between the parents to obtain the children.

The elements $a_{73}$ to $a_{88}$ of Parent 1 are exchanged with $b_{73}$ to $b_{88}$ of Parent 2 to generate children denoted by ‘Child 1’ and ‘Child 2’, respectively (Figure 11).

### 4.4. Mutation

The mutation is an important operation to preserve the genetic diversity of a population of chromosomes in every generation. The mutation is performed by exchanging one or more genes of the chromosomes. Generally, a certain percentage of the population is allowed to undergo mutation. The mutation may change the solution considerably from the previous solution. Hence, GA can arrive at a better solution by using mutations. The process for this mutation is given in Algorithm 3.
**Algorithm 3**Mutation().$m_{1}\leftarrow$ random integer [N+1,N+m]$m_{2}\left(\neq m_{1}\right)\leftarrow$ random integer [N+1,N+m]**if**$m_{1}\neq m_{2}$**then**  Swap $m_{1}^{th}$ and $m_{2}^{th}$ rows of a chromosome**end if**

**Figure 12:**
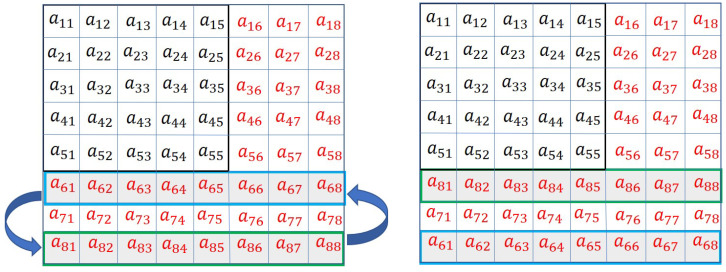
Horizontal swapping mutation.

Mutation() function is given in Algorithm 3. It swaps $m_{1}^{th}$ and $m_{2}^{th}$ rows of a chromosome. The pictorial representation of the operation is given in Figure 12. The algorithm is explained with the help of an example. Let us consider, $m_{1}=6$, and $m_{2}=8$. The selected rows are shown in green and blue lines, respectively. According to the algorithm, the sixth and eighth rows are swapped, as shown in the figure.

## 5. Results

In this study, an example scenario is considered. The existing MASs consist of ten agents, and they are assigned a mission. Depending on the requirement, more agents need to be added to the existing group of agents. All the agents have the same dynamics, i.e., they are homogeneous in nature. The nonlinear dynamics considered for the ith agents is given by

(14) $$\begin{array}{ccc} {\dot{X}}_{i_{1}} & = & X_{i_{2}}\sin\left(2X_{i_{1}}\right)+U_{i_{1}} \end{array}$$

(15) $$\begin{array}{ccc} {\dot{X}}_{i_{2}} & = & X_{i_{1}}\cos\left(3X_{i_{2}}\right)+U_{i_{2}} \end{array}$$

where $X_{i}={\left[X_{i_{1}}\;X_{i_{2}}\right]}^{T}$ and $U_{i}={\left[U_{i_{1}}\;U_{i_{2}}\right]}^{T}$ are states and control variables, respectively. The simulation study is presented in two parts. In the first part, we show the details of the existing topology. In the second part, we will show how more agents are added to the existing agents without changing the existing topology.

### 5.1. Part I: Existing Topology

**Figure 13:**
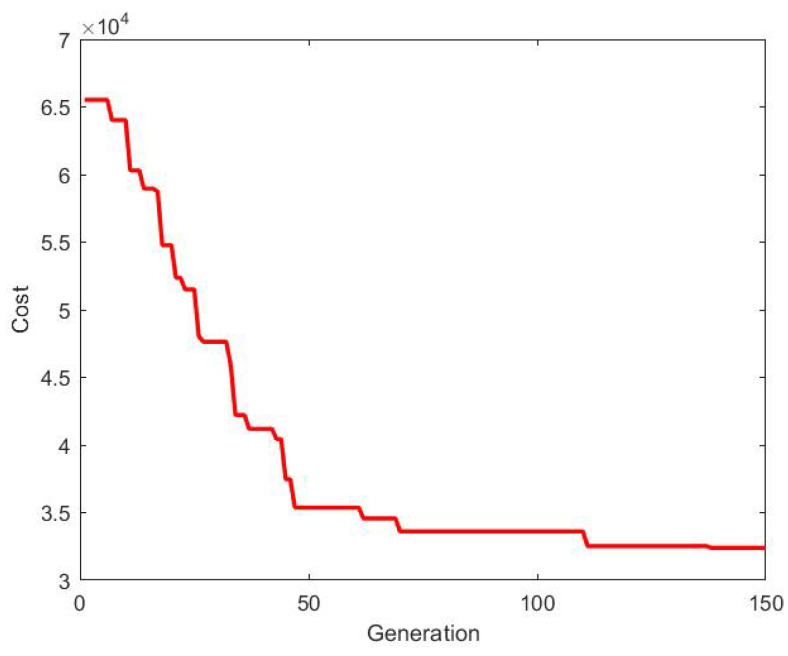
Cost for building initial optimal topology.

The existing topology in which the group of agents are connected is considered to be the optimal one [20]. The optimal topology is obtained using 2D-GA, as described in this paper. The cost (consensus control energy) generated is shown in Figure 13.

**Figure 14:**
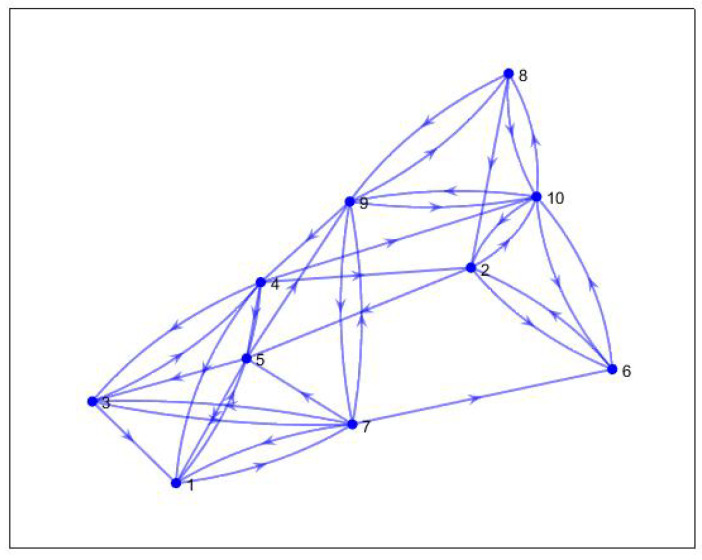
Optimal topology.

**Figure 15:**
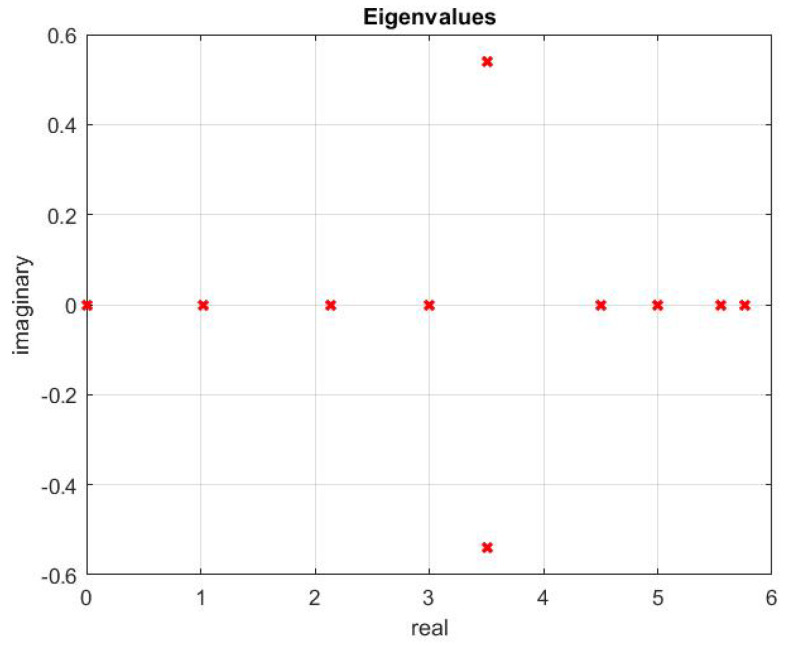
Eigenvalues.

The optimal topology obtained is shown in Figure 14. This is the existing topology among the agents. The topology contains a spanning tree, which is evident from the eigenvalues shown in Figure 15. There is only one zero eigenvalue. Others have a positive real part.

**Figure 16:**
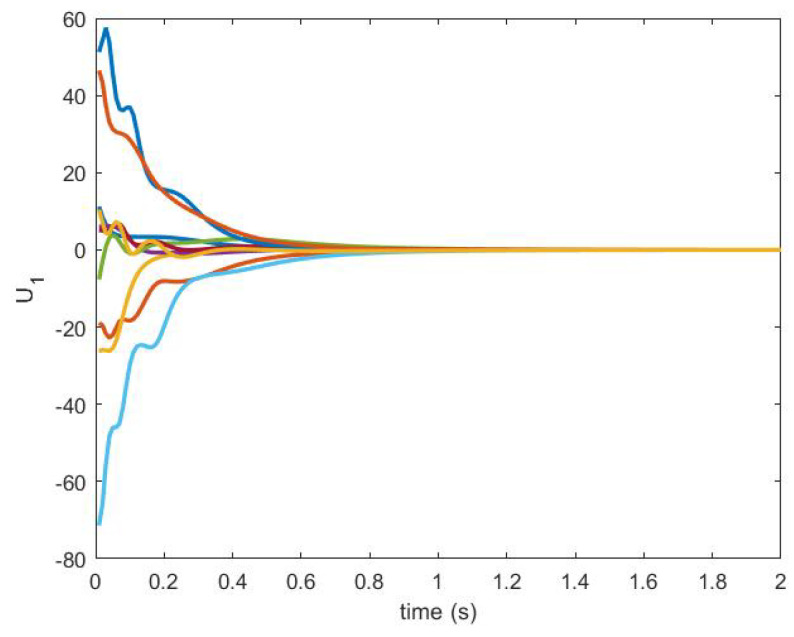
Control $U_{1}$ produced by an NDI-based controller.

**Figure 17:**
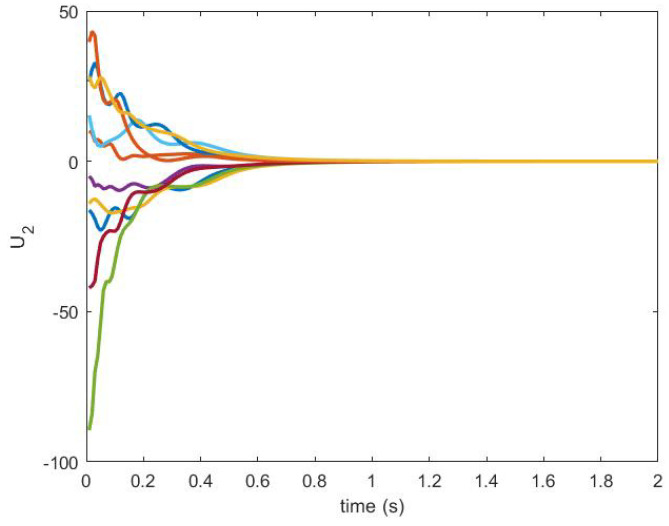
Control $U_{2}$ produced by an NDI-based controller.

The consensus is achieved by using the NDI-based controller. The control signals $U_{1}$ and $U_{2}$ are shown in Figure 16 and Figure 17, respectively.

**Figure 18:**
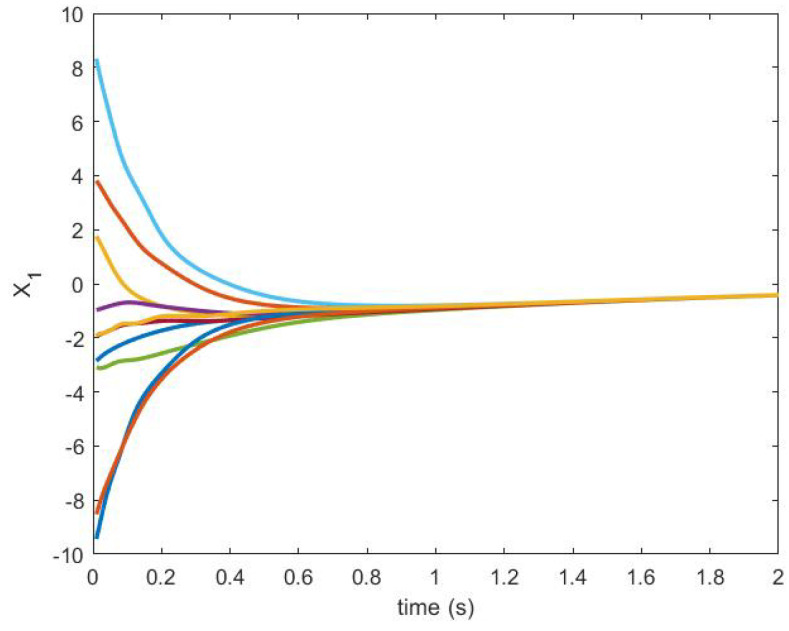
State trajectory $X_{1}$.

**Figure 19:**
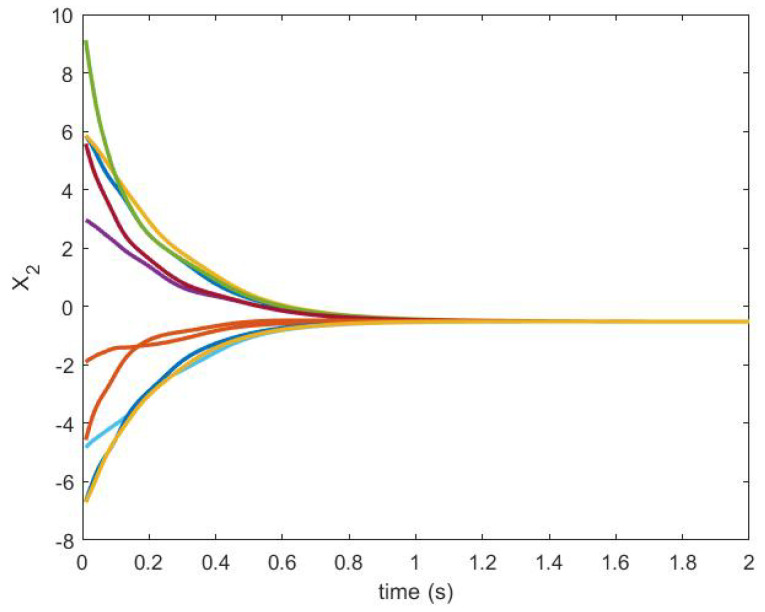
State trajectory $X_{2}$.

The trajectories generated are shown in Figure 18 and Figure 19.

The consensus among the agents is achieved successfully on this topology.

### 5.2. Part II: Agent Addition, No Change in Existing Topology

**Figure 20:**
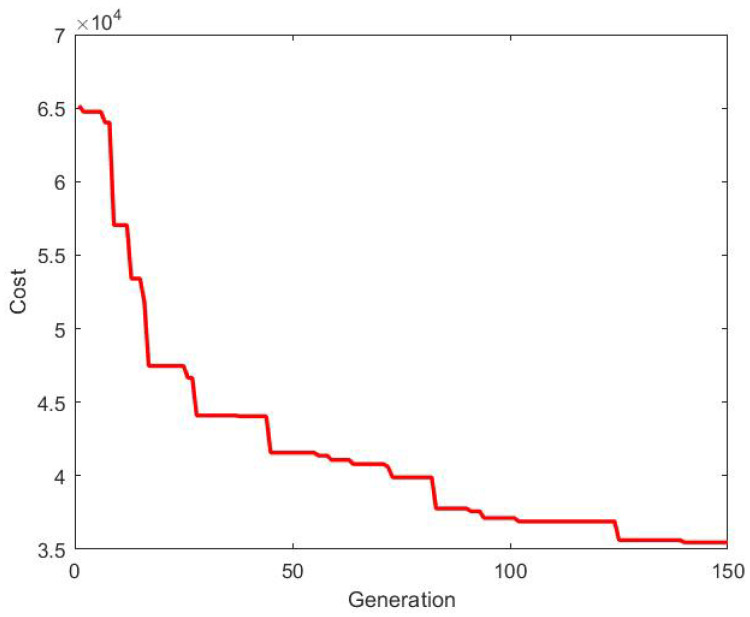
Updated cost.

In this part, the new agents are added to the existing group of agents. The existing topology is kept unchanged. The updated topology is obtained by minimizing the consensus control energy of all the agents (including new agents) using 2D-GA. The cost obtained is shown in Figure 20.

**Figure 21:**
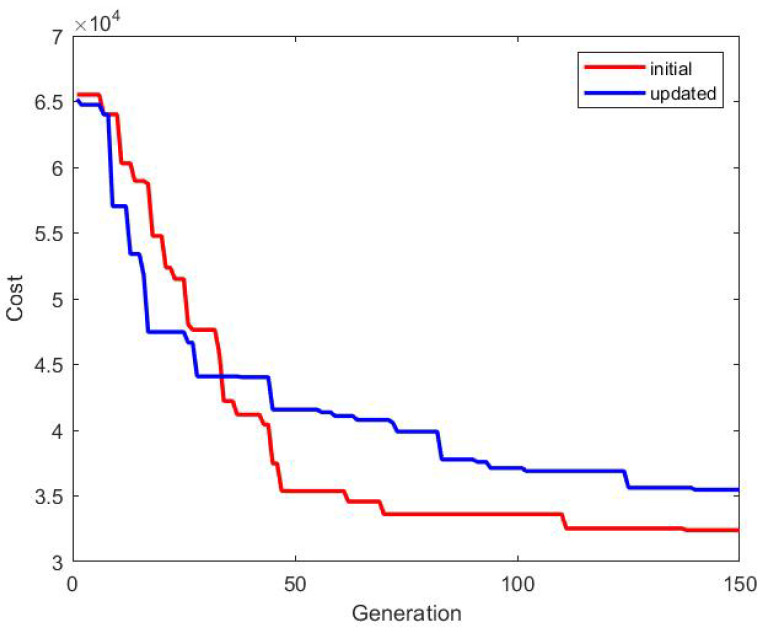
Comparison of costs between existing and updated topology.

**Figure 22:**
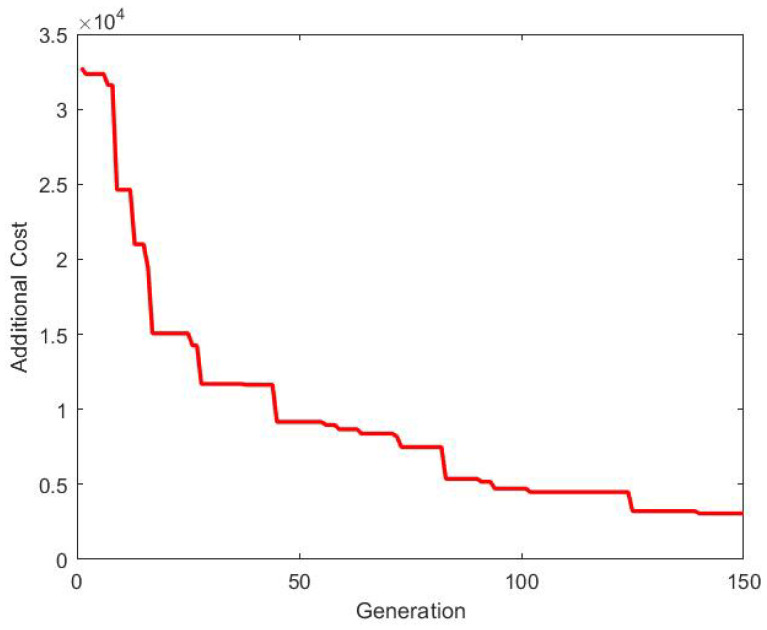
Additional cost.

It is obvious that the addition of new agents result in an increase in the consensus control energy. The objective of this paper is to minimize the additional control energy. The cost produced by 2D-GA to obtain the updated topology is compared with that of the existing topology in Figure 21. Also, the additional cost required to obtain the updated topology is shown in Figure 22.

**Figure 23:**
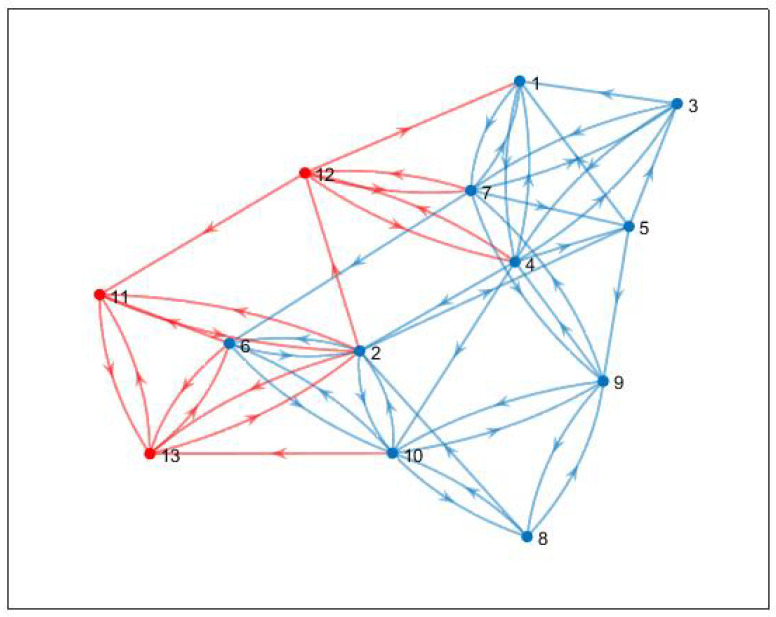
Updated topology, new agents are connected to existing agents via red edges.

It can be observed that the additional cost is minimized over the generations, i.e., the consensus of all the agents (including the new agents) can be achieved by spending the minimum amount of additional control energy. The updated topology is shown in Figure 23. The agents 11–13 are added to the existing agents in the updated topology. It is important to note that the existing topology (Figure 16) is un changed.

**Figure 24:**
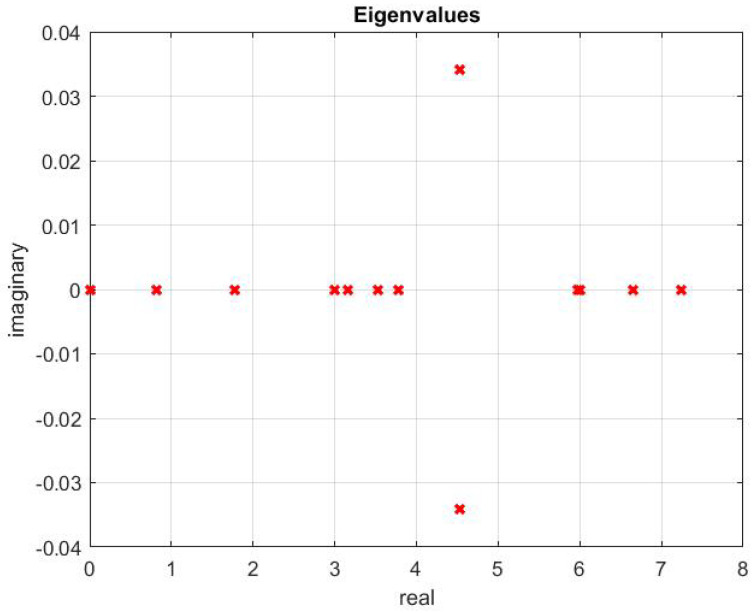
Updated topology.

The new connections are shown by red edges. The stability of this is assured by the eigenvalues shown in Figure 24, which explains the presence of a spanning tree in the topology. The eigenvalues of the updated topology are different from those corresponding to existing topology (Figure 16).

**Figure 25:**
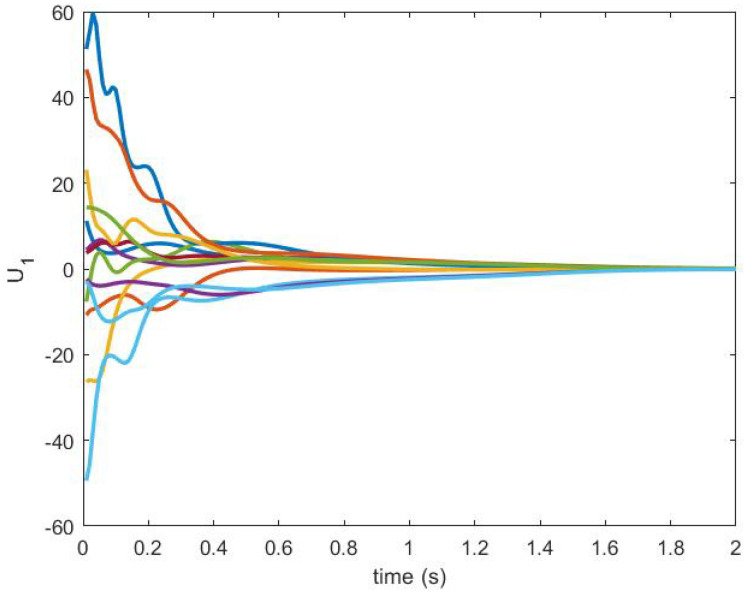
Control $U_{1}$ produced by an NDI-based controller.

**Figure 26:**
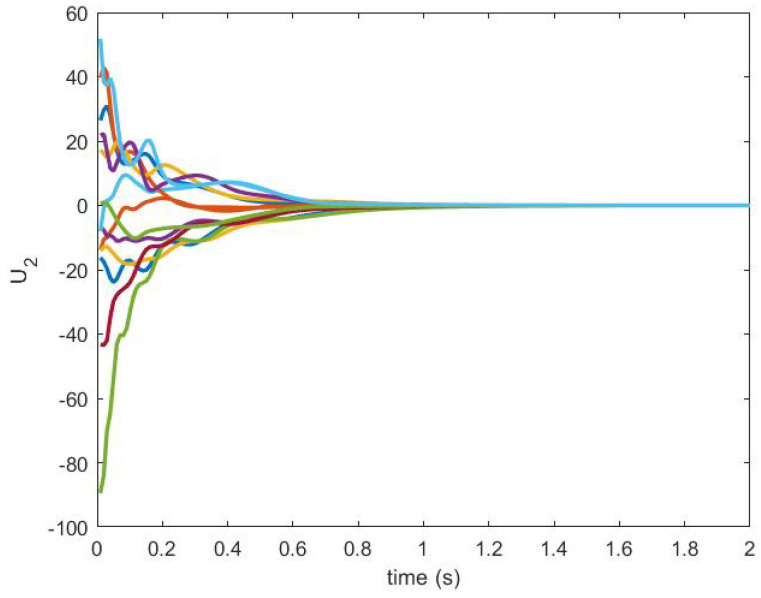
Control $U_{2}$ produced by NDI based controller.

The control signals are produced by an NDI-based controller and they are shown in Figure 25 and Figure 26.

**Figure 27:**
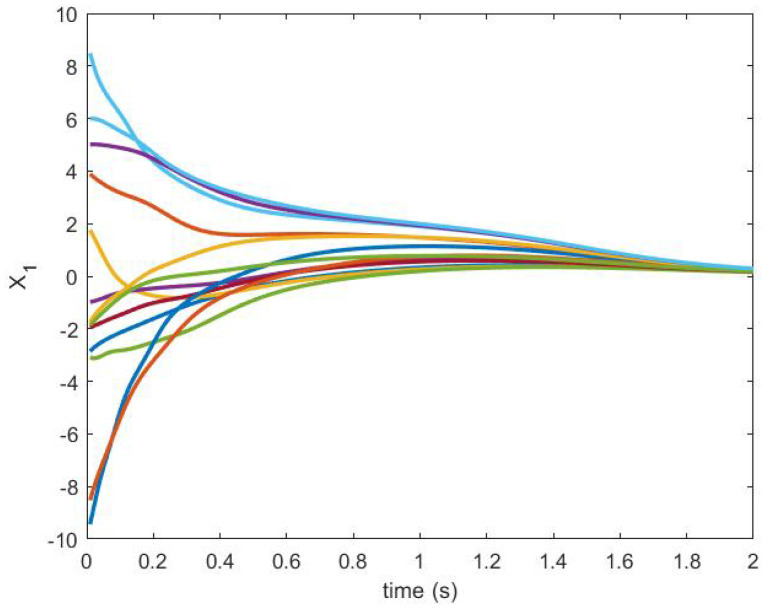
State trajectory $X_{1}$.

**Figure 28:**
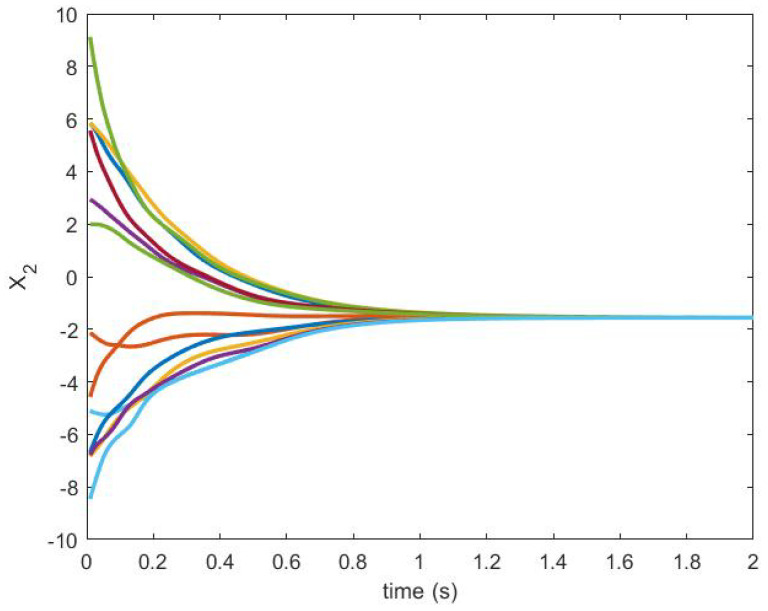
State trajectory $X_{2}$.

The state trajectories of the agents are shown in Figure 27 and Figure 28.

## 6. Conclusions

The addition of agents is achieved successfully using the proposed idea. This idea will help to build an autonomous drone platform, which is an essential requirement for BVLOS operation. The problem is addressed using a bio-inspired optimization technique which is new in the context of designing topology related to MASs. The controller based on NDI worked well for nonlinear agents. Overall, the results obtained are convincing, and they can be used for executing autonomous operations in many critical missions.
